# Comparative Genomics and Mutational Analysis Reveals a Novel XoxF-Utilizing Methylotroph in the Roseobacter Group Isolated From the Marine Environment

**DOI:** 10.3389/fmicb.2018.00766

**Published:** 2018-04-27

**Authors:** Alexandra M. Howat, John Vollmers, Martin Taubert, Carolina Grob, Joanna L. Dixon, Jonathan D. Todd, Yin Chen, Anne-Kristin Kaster, J. C. Murrell

**Affiliations:** ^1^School of Environmental Sciences, University of East Anglia, Norwich, United Kingdom; ^2^Institute for Biological Interfaces 5 (IBG-5), Karlsruhe Institute of Technology, Karlsruhe, Germany; ^3^Aquatic Geomicrobiology, Institute of Biodiversity, Friedrich Schiller University Jena, Jena, Germany; ^4^Plymouth Marine Laboratory, Plymouth, United Kingdom; ^5^School of Biological Sciences, University of East Anglia, Norwich, United Kingdom; ^6^School of Life Sciences, University of Warwick, Coventry, United Kingdom

**Keywords:** methylotrophy, *xoxF*, marine environment, Roseobacter, comparative genomics, methanol, methanol dehydrogenase

## Abstract

The Roseobacter group comprises a significant group of marine bacteria which are involved in global carbon and sulfur cycles. Some members are methylotrophs, using one-carbon compounds as a carbon and energy source. It has recently been shown that methylotrophs generally require a rare earth element when using the methanol dehydrogenase enzyme XoxF for growth on methanol. Addition of lanthanum to methanol enrichments of coastal seawater facilitated the isolation of a novel methylotroph in the Roseobacter group: *Marinibacterium anthonyi* strain La 6. Mutation of *xoxF5* revealed the essential nature of this gene during growth on methanol and ethanol. Physiological characterization demonstrated the metabolic versatility of this strain. Genome sequencing revealed that strain La 6 has the largest genome of all Roseobacter group members sequenced to date, at 7.18 Mbp. Multilocus sequence analysis (MLSA) showed that whilst it displays the highest core gene sequence similarity with subgroup 1 of the Roseobacter group, it shares very little of its pangenome, suggesting unique genetic adaptations. This research revealed that the addition of lanthanides to isolation procedures was key to cultivating novel XoxF-utilizing methylotrophs from the marine environment, whilst genome sequencing and MLSA provided insights into their potential genetic adaptations and relationship to the wider community.

## Introduction

Previous research has shown that methanol in the oceans can reach concentrations of up to 420 nM (Williams et al., 2004; Kameyama et al., 2010; Beale et al., 2011, 2013; Dixon et al., 2011, 2013a; Read et al., 2012). There has long been a debate as to whether the ocean is a source or sink of methanol, however, it has recently been revealed that various phytoplankton in laboratory cell cultures produce substantial concentrations of methanol (0.8–13.7 μM) (Mincer and Aicher, 2016). Based on these data it was estimated that phytoplankton could be the largest global source of methanol, far exceeding terrestrial plant emissions. Given the availability of methanol in the oceans, it is not surprising that some marine bacteria are able to degrade it. Methylotrophic bacteria can use one-carbon compounds, such as methanol, as a carbon and energy source (reviewed in Anthony, 1982; Chistoserdova et al., 2009; Chistoserdova, 2011). The first step in methanol oxidation is catalyzed by methanol dehydrogenases (MDH). The best characterized MDH is the Ca^2+^ containing periplasmic pyrroloquinoline quinone (PQQ)-dependent MDH found in Gram negative methylotrophs, which is an α_2_β_2_ protein encoded by *mxaF* and *mxaI* (Anthony, 1986; Chistoserdova, 2011). A second type of methanol dehydrogenase (XoxF) encoded by a homolog of *mxaF*, *xoxF*, has been discovered in many methylotrophs (Chistoserdova and Lidstrom, 1997; Giovannoni et al., 2008; Chistoserdova, 2011; Keltjens et al., 2014). This MDH is phylogenetically very diverse. With five clades (named *xoxF1*-*5*) and often multiple gene copies present, it is generally difficult to examine the exact role in methylotrophs of MDH enzymes encoded by *xoxF* (Chistoserdova, 2011; Keltjens et al., 2014).

Knowledge of marine methylotrophs has arisen from their isolation and characterization (Yamamoto et al., 1978; Strand and Lidstrom, 1984; Janvier et al., 1985; Schaefer et al., 2002; Giovannoni et al., 2008) and through the use of functional gene probing (McDonald and Murrell, 1997; Neufeld et al., 2007). For example, using *mxaF* primers, Dixon et al. (2013b) identified methylotrophs such as *Methylophaga* sp., *Burkholderiales*, *Methylococcaceae* sp., *Paracoccus denitrificans*, *Methylophilus methylotrophus*, *Hyphomicrobium* sp., and *Methylosulfonomonas methylovora* in open Atlantic waters. Active marine methylotrophs have been found to be associated with phytoplankton blooms in the English Channel (Neufeld et al., 2008a), and uncultivated *Methylophaga* have been identified after enrichments with ^13^C-labeled methanol or methylamine in DNA sable isotope probing (DNA-SIP) experiments using seawater from the same location (Neufeld et al., 2007, 2008b; Grob et al., 2015).

Marine bacteria of the Roseobacter group often comprise over 20% of the total bacterial community in coastal environments, and play key roles in the global carbon and sulfur cycles (Buchan et al., 2005; Wagner-Döbler and Biebl, 2006; Pradella et al., 2010). Many strains are associated with phytoplankton (Gonzalez et al., 2000; Grossart et al., 2005; Amin et al., 2012, 2015) and some are known to utilize one-carbon compounds (Gonzalez et al., 1997; Schäfer et al., 2005; Sun et al., 2010). For example, the methylotroph *Marinovum algicola* was isolated from the dinoflagellate *Prorocentrum lima* (Lafay et al., 1995). Hence, it is possible that such close associations are due to the ability of some Roseobacter group members to use methanol and/or other one-carbon compounds excreted by phytoplankton as carbon and energy sources. Moreover, amplicon sequencing of *xoxF* genes from clade 5 (*xoxF5)* amplified from different coastal sites (Taubert et al., 2015) revealed high relative abundances of sequences from the *Rhodobacteraceae* family such as *Sagittula* (a known marine methylotroph), but also of many unclassified *Rhodobacteraceae* sequences, supporting the hypothesis that many members of the Roseobacter group are capable of methylotrophy *in situ.* It is therefore important that the methylotrophic abilities of the marine Roseobacter group is re-examined (Martens et al., 2006; Pradella et al., 2010).

Recent research has revealed the importance of rare earth elements (REEs) such as the lanthanides cerium and lanthanum during the growth of XoxF-utilizing methylotrophs (Keltjens et al., 2014; Farhan Ul-Haque et al., 2015; Chistoserdova, 2016; Vu et al., 2016). Not only have these lanthanides been shown to be present at the catalytic site of XoxF, but they are also involved in the up-regulation of the expression of *xoxF* and down-regulation of the expression of the *mxaFI* genes encoding the classic MDH (Nakagawa et al., 2012; Keltjens et al., 2014; Pol et al., 2014; Bogart et al., 2015; Farhan Ul-Haque et al., 2015; Wu et al., 2015).

Rare earth elements are highly insoluble and are rarely found in pure form (Hu et al., 2004) and due to the relative difficulty in quantifying REEs, they are not usually measured during environmental sampling. Studies have shown that concentrations can range from high nM in estuarine and coastal environments (Elderfield et al., 1990; Hatje et al., 2014) to pM concentrations in open oceans (Greaves et al., 1991; Garcia-Solsona et al., 2014). However, very little is known about the bioavailability of REEs in the marine environment. The REE-specific *xoxF* gene is found in the genomes of a broad range of bacteria and is widely distributed throughout marine environments (Taubert et al., 2015; Chistoserdova, 2016). It is clear, therefore, that the routine addition of REEs to enrichments is vital in capturing and isolating new methylotrophs. Here we report on the isolation of a novel methylotrophic Roseobacter (strain La 6) from lanthanum-supplemented enrichments containing methanol and seawater from the coast of Plymouth, United Kingdom. The methylotrophic nature of this strain was further characterized, and the genome sequenced and compared to other members of the Roseobacter group.

## Materials and Methods

### Strains, Plasmids, and Culture Conditions

Strains and plasmids used in this study are listed in Supplementary Table 1. Strain La 6 was maintained on Marine Broth 2216 (Difco, MB) (1.5% agar) or Marine Basal Medium (MBM) with 5 mM carbon source and grown at 25°C unless otherwise stated. *Escherichia coli* was grown at 37°C on Luria-Bertani (LB) (Sambrook and Russell, 2001). Antibiotics were used at the following concentrations (μg ml^-1^): kanamycin (20), gentamicin (10) and rifampicin (20), unless otherwise stated. All carbon sources were added at 5 mM and lanthanides at 5 μM.

### Lanthanide Experiments and Isolation of Strain La 6

Seawater used for all experiments was collected from station L4 of the Western Channel Observatory, Plymouth, United Kingdom (50°15.0′ N; 4°13.0′ W). For lanthanide addition experiments, triplicate gas-tight 2 L bottles were filled with 0.75 L of surface seawater, with the addition of 0.1% marine ammonium mineral salts (MAMSs) medium (Goodwin et al., 2001), 5 mM methanol and either 5 μM lanthanum, cerium, both, or no metals (added as chloride heptahydrate salts). Enrichments were incubated at 25°C in a shaking incubator (50 rpm) and the methanol headspace concentration was monitored by gas chromatography as a proxy for methanol consumption in the liquid phase (Methods described in Supplementary Information).

Strain La 6 was isolated in October 2014 using the same experimental set up as the lanthanide addition experiments, with only lanthanum as the added metal. Enrichments were incubated for 5 days, serial dilutions of this enrichment were then plated onto MBM medium containing lanthanum and incubated with methanol in the headspace of a gas tight chamber for 8 days. Colonies were re-streaked to purify and growth on methanol was confirmed by inoculation into liquid MBM containing methanol and lanthanum. Methods for physiological characterization of the strain can be found in the Supplementary Information.

### Genetic Manipulations

A single allelic exchange method was used to generate an insertional mutation in the *xoxF* gene of *Marinibacterium* sp. La 6 (Todd et al., 2011). A 672 bp internal fragment of the *xoxF* gene was amplified by polymerase chain reaction (PCR), ligated into the suicide vector pK19mob (Schäfer et al., 1994) to form p672*xoxF* and transformed into *E. coli*. Plasmid p672*xoxF* was conjugated into strain La 6^Rif^, a spontaneous rifampicin-resistant mutant, in triparental matings with helper plasmid pRK2013 (Figurski and Helinski, 1979). Rif^R^ and Kan^R^ single cross over transformants were checked using colony PCR with primers that amplified a region spanning from within the disrupted genomic *xoxF* gene to inside the kanamycin cassette of the incorporated p672*xoxF* plasmid (Supplementary Table 1). The mutant strain was termed La 6 XoxF^-^. To complement strain XoxF^-^, the complete *xoxF* sequence was amplified by PCR, ligated into the broad host range vector pLMB509 (Tett et al., 2012) and transformed into *E. coli*. Transconjugants were screened using the primers that were used to originally amplify the *xoxF* gene and the insert was then sequenced. The confirmed plasmid was termed p509LA6. This plasmid was then conjugated into La 6^Rif^ using triparental matings, and the resulting complemented strain was termed La 6 XoxF^-^ p509LA6.

### Genome Sequencing, Assembly, and Annotation

Genomic DNA was extracted using the CTAB (cetyl-trimethylammonium bromide) method of Doyle and Doyle (1987). The genome of strain La 6 was sequenced as follows: standard and mate-pair sequencing libraries were produced using Illumina kits and run on a Miseq machine using V3 chemistry with a paired-end approach and 301 cycles per read. Reads were adapter-clipped and quality trimmed using Trimmomatic (Bolger et al., 2014). Mate-pair reads were additionally clipped, sorted and re-orientated using NxTrim (O’Connell et al., 2015). Potential PhiX and vector contamination were filtered out using fastq_screen^1^, while low complexity reads (consisting entirely of only one base type or direct short oligonucleotide repeats) were removed using prinseq (Schmieder and Edwards, 2011). Potential overlapping paired-end reads were merged using FLASH (Magoč and Salzberg, 2011). Assembly was done using Spades v.3.8. ORF-calling and annotation were done using the PROKKA pipeline v.1.12 (Seemann, 2014). The draft genome sequence of strain La 6 is available in GenBank under Accession No. NSDV00000000; the strain deposit number is DSM 104755.

### Comparative Genomics

For multilocus sequence analysis (MLSA), the unique core genome of 94 comparison genomes (including *Parvularcula bermudensis* HTCC2503 as the outgroup) consisting of 219 gene products with a combined length of 95,680 amino acid residues was determined using the bidirectional BLAST+ approach implemented in proteinortho5 (Lechner et al., 2011), excluding all genes with duplicates in any comparison genome. After alignment with muscle (Edgar, 2004), the gene products were concatenated and unalignable regions were filtered out using gblocks (Castresana, 2000), leaving 56,810 aligned amino acid residues for phylogenetic analysis. Clustering was performed using the Neighbor Joining algorithm with 1,000 bootstrap permutations.

For gene content analyses, a binary matrix was constructed, representing the presence or absence of orthologous groups identified by the bidirectional BLAST+ approach mentioned above. In order to prevent artifacts caused by fragmented or falsely predicted genes, all singletons were excluded from the analyses (requiring each considered orthologous group to be present in at least two different genomes). This resulting binary matrix was converted into a distance matrix and clustered using the Neighbor Joining algorithm and 1,000 bootstrap permutations.

## Results and Discussion

### Isolation of a Novel Methylotroph Using Lanthanum

Traditional methylotroph enrichment and isolation experiments using water from station L4 of the Western Channel Observatory (Plymouth, United Kingdom; 50°15.0′ N; 4°13.0′ W) not supplemented with lanthanides frequently gave rise to the isolation of *Methylophaga* sp. (Howat, 2017), whilst cultivation-independent research using DNA-SIP consistently showed that *Methylophaga* are also the dominant methylotrophs metabolizing methanol in enrichment cultures (Neufeld et al., 2007, 2008b; Grob et al., 2015). *Methylophaga* spp. contain both *mxaF* and multiple copies of *xoxF*, and while there has been no direct evidence that *Methylophaga* spp. use MxaF rather than XoxF during growth on methanol, high levels of MxaF expression have been observed when methylotrophs are grown on methanol, suggesting the use of this calcium-containing methanol dehydrogenase enzyme (Choi et al., 2011; Kim et al., 2012). However, the model methylotroph *Methylobacterium extorquens* also contains both *xoxF* and *mxaF* genes, and work on this bacterium showed that it expressed XoxF instead of MxaF when lanthanide concentrations were higher than 100 nM (Vu et al., 2016). It may be possible that the seawater used in previous methanol enrichment experiments described above did not contain sufficient concentrations of REEs to support growth of XoxF-utilizing methylotrophs. Therefore, the effect of the addition of lanthanides to seawater enrichments containing methanol was examined using surface seawater from station L4, Plymouth.

Methanol enrichments containing either 5 μM lanthanum, cerium, or both showed a significant increase in methanol depletion (*p* ≤ 0.05) compared to those without, suggesting that the bacterial oxidation of methanol was stimulated by the addition of the metals (Supplementary Figure 1). When lanthanum was then added to subsequent enrichments and isolation agar, a novel methylotroph (strain La 6) was isolated from station L4. This strain represented three out of 20 screened isolates selected for their ability to grow on methanol; all other strains being (*Methylophaga* sp.). The corresponding 16S rRNA gene sequence of the isolate was 99% identical to *Marinibacterium profundimaris* strain 22II1-22F33^T^ (Supplementary Figure 2) (Li et al., 2015). The relatively low colony counts of this isolated Roseobacter probably reflected the fact that they were a small proportion of the methylotrophs present in the seawater enrichment, however, previous research using very similar enrichment procedures gave rise to no Roseobacters at all (Howat, 2017), suggesting that the addition of lanthanum aided methylotrophic growth of Roseobacters to support a population dense enough to be subsequently isolated.

**FIGURE 1:**
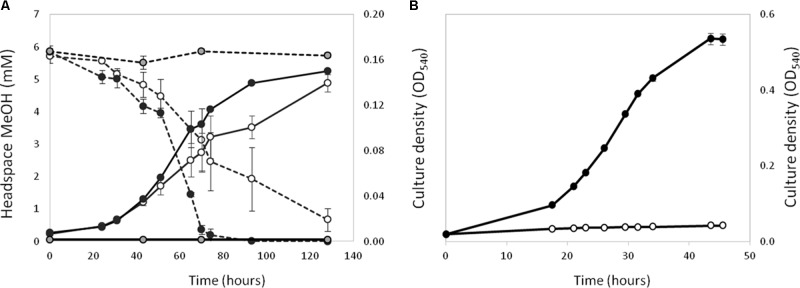
Effect of the presence (black circles) or absence (white circles) of 5 μM lanthanum on the growth (solid lines) of strain La 6 on methanol (**A**, 5 mM initial concentration) and ethanol (**B**, 5 mM initial concentration). Dotted lines represent headspace methanol concentrations. Gray circles are no-inoculum controls containing lanthanum. Error bars are the standard error of three replicates.

Polymerase chain reaction assays on genomic DNA from strain La 6 and subsequent Sanger sequencing indicated that the isolate contained only one copy of *xoxF* from clade 5 and no *mxaF* in its genome (later confirmed by genome sequencing, see below). When grown in MBM, strain La 6 exhibited lanthanum-stimulated growth on methanol, whilst there was an absolute requirement for lanthanum ions when grown on ethanol as carbon source (**Figure 1**).

*Marinibacterium profundimaris* was not previously tested for growth on methanol and its genome contained no predicted MDH. Therefore the physiology of strain La 6 was further characterized, the genome sequenced and its ability to grow methylotrophically was investigated to further understand the role of *xoxF5* in this marine strain.

### Physiological Characteristics

Strain La 6 utilized a wide range of carbon compounds including methanol, ethanol, propane, and butane (for a full list of compounds see Supplementary Table 2). Tests for growth of the strain on methanol at concentrations higher than 5 mM yielded no increase in final cell density.

Strain La 6 is a Gram negative, ovoid rod, 0.8–2.2 μm long and 0.5–1.2 μm wide when grown on minimal medium. It is non-motile when tested on swimming, swarming or twitching motility plates and in liquid medium. Colonies are very pale cream and 0.5–1.0 mm in diameter, uniformly circular, convex, and opaque after growth on MBM minimal media at 25°C for 6 days. Colonies are cream and 0.6–1.2 mm in diameter, uniformly circular, convex, and opaque after growth on marine agar 2216 at 25°C for 4 days.

Temperature range for growth was 4–45°C, with the optimum at 37°C. The pH range for growth was pH 4.5–9 (optimum 7.5) and the NaCl concentrations for growth were 0–15% w/v (optimum 3%), with no growth at 20%. It did not grow under anaerobic conditions and did not reduce either nitrate or nitrite. It did not hydrolyse cellulose, gelatine or starch, nor did it ferment glucose or lactose aerobically or anaerobically. Strain La 6 was negative for thiosulfate oxidation. It produced indoleacetic acid when supplemented with tryptophan, but not without. Strain La 6 did not produce any acetone/methanol extractable pigments or bacteriochlorophyll *a* after growth in either a light/dark cycle or in the dark after 5 days at 22°C, therefore suggesting growth of the isolate is exclusively chemoheterotrophic and non-photosynthetic. Strain La 6 required vitamin B_12_ for growth, and was oxidase and catalase positive. Like many of the family of the *Rhodobacteraceae*, the principle fatty acid composition was 18:1ω7c (67.83%) and had a fairly similar profile to *M. profundimaris* 22II1-22F33^T^, however, it can be differentiated by the presence of summed feature 2 (14:0 3-OH/16:1) (7.31%) (Supplementary Table 3).

### Genome Sequencing and Genome Analysis of Strain La 6

Sequencing of the genome of strain La 6 yielded 15 contigs covering a total length of 7.2 Mbp (mol % GC content 65.4). Based on sequence similarities, 73% of protein-coding genes could be assigned a putative function, whilst one quarter of them were classified as ‘hypothetical,’ using the software tool PROKKA (Seemann, 2014) (full genome statistics are summarized in **Table 1**). Assessment of the genome quality using CheckM (Parks et al., 2015) yielded a ‘completeness’ value of 99.41%, which is above the average value of 99.1% found in the currently published Roseobacter group genomes, indicating complete genome reconstruction (Supplementary Table 4). The genome suggested a complete tricarboxylic acid cycle (TCA) pathway and genes for the pentose phosphate pathway, Entner-Doudoroff and Embden-Meyerhof pathways. It contained all genes required for ammonia assimilation (including glutamate dehydrogenase, glutamine synthetase, glutamine oxoglutarate amidotransferase, and alanine dehydrogenase) and those encoding nitrogenase; it did not contain genes encoding ribulose-1,5-bisphosphate carboxylase/oxygenase.

**Table 1:** Genome statistics of strain La 6 compared to *M. profundimaris* strain 22II1-22F33^T^.

Genome data	Strain La 6	*M. profundimaris*
Genome size (bp)	7,179,825	6,152,202
GC content (%)	65.4	66.2
Number of contigs	15	60
Smallest contig (bp)	948	580
Largest contig (bp)	3,672,580	1,058,968
Average contig size (bp)	478,655	102,536
Median contig size (bp)	103, 981	35,546
N50	3,672,580	343,537
L50	1	5
Number of genes	6,844	5,628
Number of coding sequences (% of homologs with closest strain)	6,785 (64%^∗^)	5,497 (74%^∗∗^)
Number of hypothetical proteins (%)	1,835 (27)	985 (18%)
tRNAs	52	44
rRNAs	6	4

*^∗^% of the protein coding genes in La 6 that have a homolog in M. profundimaris. ^∗∗^% of the protein coding genes in M. profundimaris that have a homolog in strain La 6.*

### Genome-Inferred Methylotrophic Pathways in Strain La 6

**FIGURE 2:**
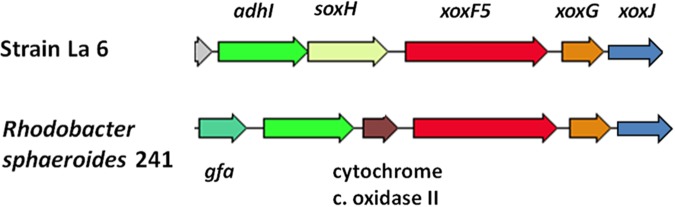
Gene cluster surrounding the predicted methanol dehydrogenase gene *xoxF5* and comparison to the methylotroph *Rhodobacter sphaeroides* 241. Colors indicate predicted similar functions of genes between the two organisms. *adhI*, glutathione-dependent formaldehyde dehydrogenase; *soxH*, putative protein SoxH; *xoxF5*, methanol dehydrogenase; *xoxG*, cytochrome c-553i; *xoxJ*, hypothetical periplasmic binding protein; *gfa*, homolog of glutathione-formaldehyde activating enzyme; cytochrome c oxidase II.

Genome sequencing confirmed that *xoxF* from clade 5 (*xoxF5*, one copy) was the only predicted MDH-encoding gene in the genome of strain La 6, and that it was adjacent to *xoxG* (encoding an associated cytochrome c used as an electron acceptor during methanol oxidation) and *xoxJ*, encoding a putative periplasmic binding protein (Chistoserdova, 2011). Adjacent genes were similar to those found in the known methylotrophs *Rhodobacter sphaeroides* and *Paracoccus aminophilus* JCM7686, that employ the glutathione-dependent formaldehyde oxidation pathway (Wilson et al., 2008; Dziewit et al., 2015) and only contain *xoxF5* (**Figure 2**).

**FIGURE 3:**
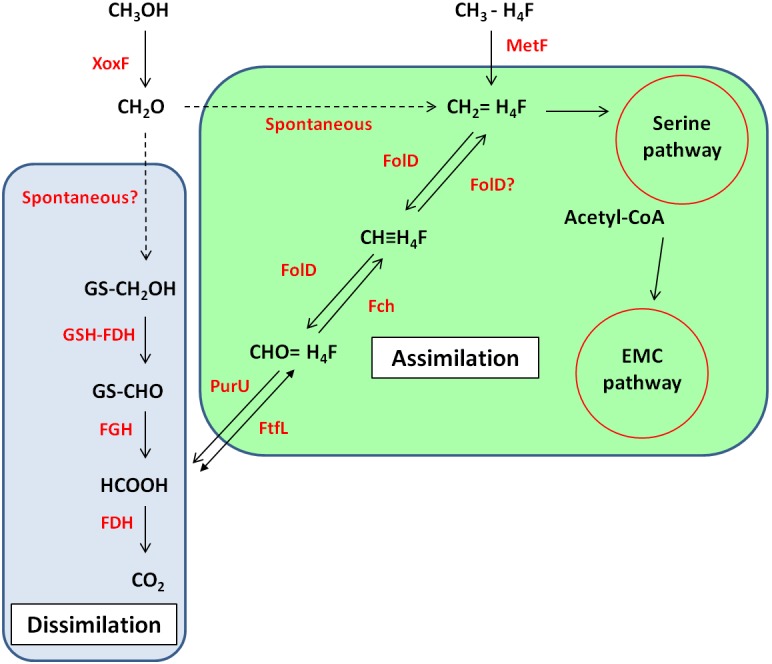
Predicted metabolic pathway of methanol metabolism in strain La 6 based on genome sequence analysis. Enzymes are shown in red whilst compounds and names of pathways are in black. Solid arrows indicate enzymatic reactions, dashed arrows indicate reactions are non-enzymatic or unknown. Reactions within the blue/green box are part of the dissimilatory/assimilatory pathway. XoxF, methanol dehydrogenase; GSH-FDH, glutathione-dependent formaldehyde dehydrogenase; FGH, *S*-formylglutathione hydrolase; FDH, formate dehydrogenase; PurU, 10-formyl-H_4_F hydrolase; FtfL, formyl-H_4_F ligase; FolD, bifunctional methylene-H_4_F dehydrogenase- methenyl-H_4_F cyclohydrolase; Fch, methenyl-H_4_F cyclohydrolase; MetF, methyl-H_4_F reductase; EMC, ethylmalonyl-CoA; PHB, polyhydroxybutyrate.

In *R. sphaeroides*, the formaldehyde produced by XoxF is initially converted to *S*-hydroxymethyl-gluthathione (GS-CH_2_OH) by a glutathione-formaldehyde activating enzyme (Gfa) or by a spontaneous reaction. This is then further oxidized by other enzymes to CO_2_ to generate energy (Wilson et al., 2008). However, unlike *R. sphaeroides*, the gene cluster around *xoxF5* of strain La 6 does not contain *gfa* (see **Figure 2**). BLAST searches of the genome using the Gfa from *R. sphaeroides* revealed some candidates, however none were more than 35% identical at the amino acid level. Searches for a formaldehyde activating enzyme gene, *fae*, which is used in other organisms revealed no candidates either (Vorholt et al., 2000). It is possible, therefore, that strain La 6 either does not contain a gene responsible for converting formaldehyde to GS-CH_2_OH, relying solely on a spontaneous chemical reaction, or it has an as yet-unidentified mechanism (**Figure 3**).

La 6 contained *gmaS*, a key gene of the N-methylglutamate pathway for methylamine metabolism. It did not contain, *mauA*, the gene encoding for a subunit of an alternative methylamine degrading enzyme, methylamine dehydrogenase. However, the strain was unable to grow on methylamine as a carbon and energy source (Supplementary Information). Lastly, strain La 6 also contains the gene encoding methyl-H_4_F reductase (MetF) which oxidizes methyl-H_4_F originating from demethylation reactions such as in the metabolism of dimethylsulfoniopropionate (DMSP) or chloromethane (Studer et al., 2001, 2002; Reisch et al., 2008; Curson et al., 2011). However, strain La 6 did not contain the *cmuAB* or *dmdA* genes that would suggest metabolism of chloromethane or DMSP (further discussed below).

For carbon assimilation, the genome of strain La 6 contains all the genes of the tetrahydrofolate-linked (H_4_F) pathway. This pathway generates the key metabolite methylene-H_4_F, which can either feed into the serine cycle for assimilation or serve as a further source of formate for generating energy (Chistoserdova, 2011). In strain La 6, this pathway may either rely on the spontaneous reaction between formaldehyde and H_4_F or it may also be possible that FolD (bifunctional methylene-H_4_F dehydrogenase-methenyl-H_4_F cyclohydrolase) can function in the reductive direction and generate methylene-H_4_F for assimilation (Chistoserdova, 2011). Formate generated through the glutathione-linked pathway could be fed *via* the reversible enzyme formyl-H_4_F ligase (FtfL) and methenyl-H_4_F cyclohydrolase (Fch) onto FolD. The genome of strain La 6 also contains genes encoding for three formate dehydrogenases (FDH); FDH1, 2, and 3.

Strain La 6 contained all the genes of the serine pathway. Methylotrophs utilizing the serine cycle require an additional pathway for regenerating glyoxylate; strain La 6 encodes all the genes for the ethylmalonyl-CoA pathway (EMCP) and does not contain isocitrate lyase, whilst it also had the potential to make PHB, containing the PHB synthase genes. A summary of predicted methylotrophic pathways based on the genome sequence and some physiological data is shown in **Figure 3**.

### The Role of XoxF During Growth of Strain La 6 on Methanol and Ethanol

**FIGURE 4:**
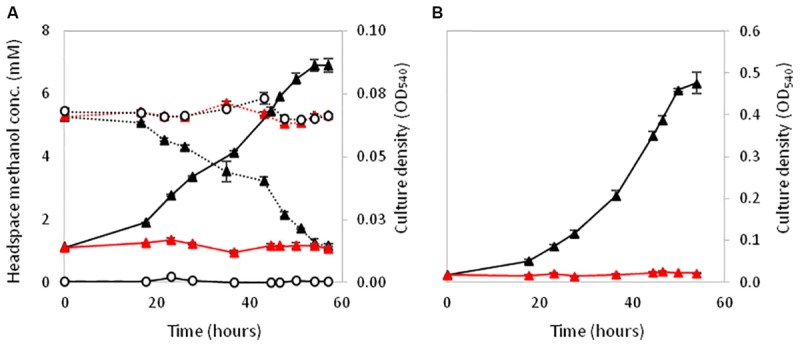
Growth of La 6 wild-type strain (black triangles), strain XoxF^-^ (red triangles) or no inoculum controls (white circles) on 5 mM methanol initial concentration **(A)** and 5 mM ethanol initial concentration **(B)**. Dashed lines in **(A)** represent methanol headspace concentrations. All conditions contained 5 μM lanthanum. Error bars show standard error of three replicate cultures.

XoxF5 is the sole MDH responsible for methanol oxidation in the two relatives of the Rosoebacter group, *R. sphaeroides* and *P. aminophilus*. However there are many Roseobacters that contain either a single *xoxF* from clade 5 but are unable to grow on methanol (or have not been tested) or the role of *xoxF5* of those that do grow on methanol was not previously examined (Shiba, 1991; Cho and Giovannoni, 2006; Lee et al., 2007; Li et al., 2015). Thus, we investigated the role of the *xoxF5* gene in strain La 6. Mutation of *xoxF5* in strain La 6 abolished the growth of the mutant strain La 6 XoxF^-^ on both methanol and ethanol (**Figure 4**). Cell-free extracts of the wild-type strain grown on methanol contained substantial methanol dehydrogenase activity (262 nmol min^-1^ mg^-1^ protein; ±6 SE). SDS-PAGE and mass spectrometry analysis of the wild-type grown on various carbon sources (methanol, ethanol, succinate, or benzoate) revealed the expression of XoxF in cells grown under all of these conditions, whilst the mutant did not express XoxF (Supplementary Figure 3). Complementation of the mutant with the wild-type *xoxF5* gene restored growth on both methanol and ethanol. SDS-PAGE analysis of cell free-extracts of this complemented *xoxF5* mutant confirmed restoration of expression of XoxF5 (Supplementary Figures 3, 4). These data confirm that *xoxF5* is directly involved in the oxidation of methanol and ethanol in strain La 6 and that XoxF5 is essential for growth on these compounds.

### Roseobacter-Specific Traits

Members of the Roseobacter group are known to grow on various aromatic and phenolic compounds (Buchan, 2001; Buchan et al., 2004; Alejandro-Marín et al., 2014). The ability of these organisms to degrade naturally occurring but potentially harmful compounds such as polycyclic aromatic hydrocarbons (PAHs) demonstrates the ecological importance of the Roseobacter group (Seo et al., 2009). When tested, strain La 6 grew on a range of aromatics, including benzoate, 4-hydroxybenzoate, protocatechuate, and catechol. Analysis of the genome revealed the presence of genes that could explain such capabilities, such as the *benABCD* cluster which encodes for benzoate dioxygenase, and the *pcaQDCHGB* cluster for protocatechuate metabolism (Buchan et al., 2004; Alejandro-Marín et al., 2014). Strain La 6 was unable to grow on toluene, *p*-cresol, *p*-xylene, 3-hydroxybenzoate, benzene, naphthalene, vanillate, or 4-chlorobenzoate.

Many Roseobacters are also able to metabolize the abundant sulfurous osmolyte DMSP, via demethylation and/or cleavage generating methanethiol or dimethylsulfide (DMS), respectively (Curson et al., 2011). DMS oxidation products in the atmosphere can act as cloud condensation nuclei, as chemo-attractants for many marine animals and are a major source of organic sulfur in the sulfur cycle (Schäfer et al., 2010; Curson et al., 2011; Moran et al., 2012). As with many Roseobacters, strain La 6 did not grow on DMSP as sole carbon source, but whole cells of strain La 6 did cleave DMSP, generating DMS at a rate of 72 nmol min^-1^ mg^-1^ protein (4.8 SE). This DMSP-dependent DMS production is probably due to expression of the DMSP lyase gene *dddL* (which has 48% identity to DddL of *Sulfitobacter* sp. EE-36) that is present in the genome of strain La 6 (Curson et al., 2011). As mentioned previously, the genome of strain La 6 lacked a *dmdA* gene homolog, which encodes the DMSP demethylase enzyme (Moran et al., 2012), which is consistent with our finding that La 6 produced no MeSH above background levels (data not shown).

Recently Curson et al., 2017 discovered that some Roseobacters, such as *Labrenzia aggregata*, can produce DMSP and contain the *dsyB* gene, which encodes the key methylthiohydroxybutyrate methyltransferase enzyme of DMSP synthesis (Curson et al., 2017). The genome of strain La 6 contained a *dsyB* homolog (73% amino acid identity to *L*. *aggregata* DsyB) and strain La 6 cell also synthesized DMSP at a rate of 2.3 nmol min^-1^ mg^-1^ protein (0.15 SE). It will be interesting to investigate why strain La 6 produces DMSP and what its intracellular function is in future studies. Some members of the Roseobacter group can also produce DMS independently of DMSP via methylation of methane-thiol, and contain the methanethiol methyltransferase enzyme termed MddA (Carrión et al., 2015). However, strain La 6 contains no MddA homolog and produced no DMS when grown in the absence of DMSP, irrespective of MeSH addition. The fact that strain La 6 produces DMSP but releases no detectable DMS in the absence of DMSP addition at high levels suggests that the DMSP lyase might only function when DMSP reaches high intracellular levels (Sun et al., 2016). Again, this aspect or organic sulfur metabolism in strain La 6 warrants further investigation in the future.

### Comparative Genomics

**FIGURE 5:**
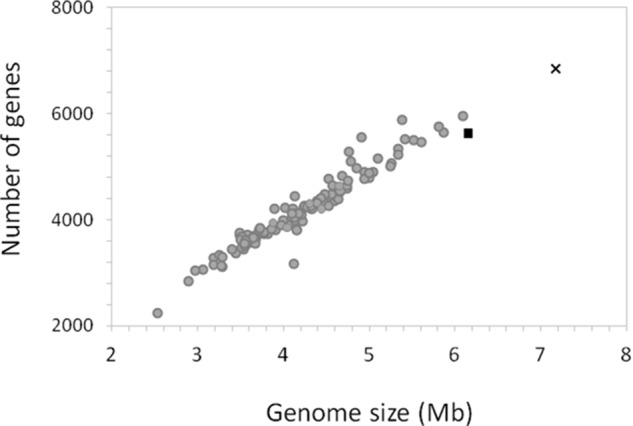
Relationship between genome size and number of genes in the genome of strain La 6 compared to the genomes of 114 members of the Roseobacter group. The genome of strain La 6 is represented by the black cross, the black triangle is the closest relative at the 16S rRNA gene sequence, *Marinibacterium profundimaris* strain 22II1-22F33^T^ and gray circles depict all other members of the Roseobacter group.

Members of the Roseobacter group are known for having large genomes, versatile metabolic capabilities and a relatively high GC contents (Luo and Moran, 2014). Strain La 6 is no exception. Indeed, it has the largest genome of all sequenced members of the Roseobacter group to date, at 7.18 Mbp, compared to the next largest genome of *M. profundimaris* strain 22II1-22F33^T^ at 6.15 Mbp (**Figure 5**). Although the high similarity of the 16S rRNA gene sequences suggests they are the same species, the estimated DNA-DNA-Hybridization (DDH) value between *M. profundimaris* 2II1-22F33^T^ and strain La 6, determined using the GGDC online tool (Meier-Kolthoff et al., 2014), is 35%. The probability for being the same species given by GGDC is <1%, therefore supporting the designation of strain La 6 as a new species within the genus *Marinibacterium.* Analyses of homologs shared between the two strains also reveal that whilst 74% of the protein coding genes of *M. profundimaris* have a homolog in strain La 6, only 64% of the protein coding genes in the genome of strain La 6 have a homolog in *M. profundimaris* (**Table 1**).

**FIGURE 6:**
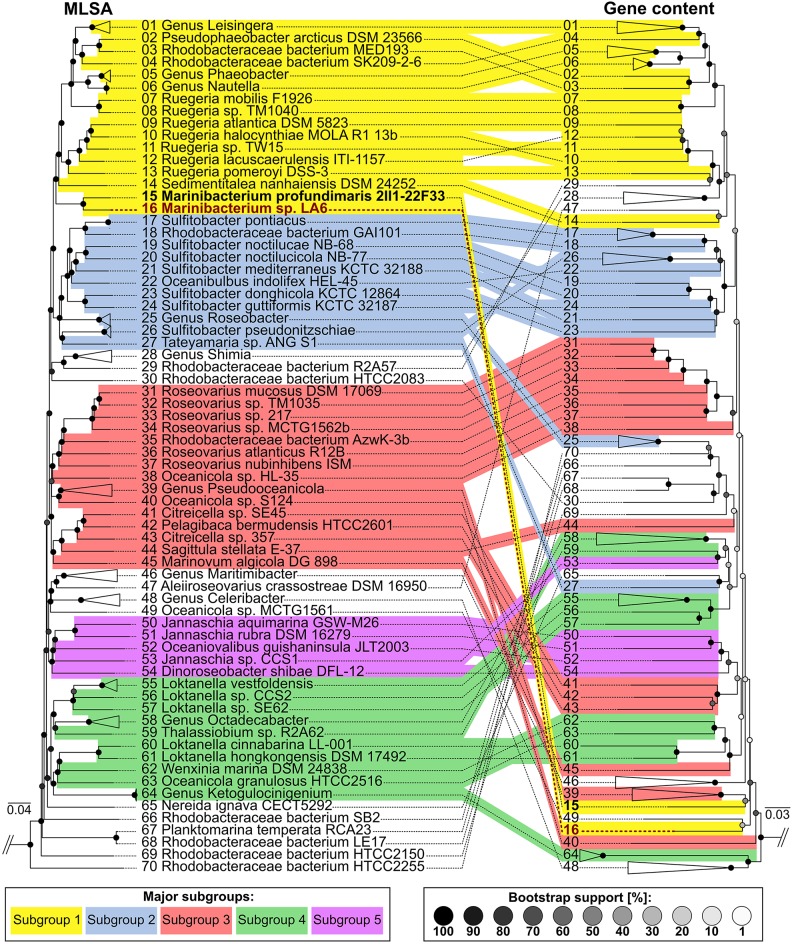
Clustering of Roseobacter group genomes showing the relationships between sequenced strains based on multilocus sequence analyses (MLSA) as well as gene content. MLSA **(left)** is based on concatenated aligned core-genome gene product sequences and illustrates phylogenetic relationships with high resolution and confidence. Coherent clusters corresponding to the five subgroups originally described by Newton et al. (2010) are marked in color. Corresponding branches between the MLSA and gene content tree are indicated by identical numbering. For ease of viewing, genera and species consisting of multiple genomes which cluster coherently in the MLSA as well as the gene content tree are shown collapsed. Furthermore, the outgroup (*Parvularcula bermudensis* HTCC2503) is not shown. In contrast, gene content clustering **(right)** is based on the presence and absence of orthologs shared between the comparison genomes. This illustrates similarities and differences in gene composition between genomes, thereby reflecting adaptations to individual niches and lifestyles. Divergences between MLSA- and gene content-based clustering show that even closely related strains may possess strongly diverging gene compositions.

Multilocus sequence analysis was performed in order to examine the phylogenetic relationship based on sequence comparisons of the unique Roseobacter core genome, with a similar topology seen from previous analyses (Buchan et al., 2005; Newton et al., 2010; Luo and Moran, 2014; Simon et al., 2017). Gene content analysis was performed and compared against the MLSA to investigate the similarities and differences in gene composition between genomes, thereby reflecting possible adaptations to individual niches and lifestyles (**Figure 6**). Overall, strain La 6 clusters deeply but coherently within subgroup 1 of the Roseobacter group, which currently consists of at least seven genera such as *Leisingera, Ruegeria*, *Sedimentitalea*, and *Marinibacterium*. However, at a gene content level, strain La 6 (and *M. profundimaris*) clusters distinctly apart from subgroup 1 and far more closely with the *Oceanicola* and *Celeribacter* genera as well as *Ketogulonicigenium vulgare*, indicating unique genetic adaptations. Bi-directional BLAST searches of all validly published Roseobacter genomes for *xoxF5* also showed that just under one fifth of all genomes harbor this gene (Supplementary Table 5).

## Conclusion

By adding lanthanides to methanol seawater enrichments, we isolated a novel member of the Roseobacter clade that can use methanol as a carbon and energy source. This isolation arose due to the discovery that upon addition of either cerium or lanthanum to methanol seawater enrichments, there was a marked increase in methanol oxidation compared to enrichments without added lanthanides. Due to the difficulty in quantifying lanthanides in marine samples, at the time of sampling it was not possible to measure the standing concentrations of these in the coastal seawater samples. However, the results do suggest that concentrations were low enough such that the addition of 5 μM lanthanide was sufficient to stimulate an increase in biological methanol oxidation.

Whilst it is known that XoxF is a lanthanide-dependent enzyme in some strains, our results from growth experiments with strain La 6 suggested that lanthanum was not strictly required for growth on methanol, only for ethanol, as there was only a slight stimulation upon addition of the metal. Contamination of lanthanides from glassware is sufficient to support the growth of some methylotrophs (Pol et al., 2014), however, this does not explain why strain La 6 was completely unable to grow on ethanol in similar levels of lanthanide ‘contaminants.’ In order to understand the catalytic mechanism of this XoxF, further work should involve purification of the enzyme from cells grown with different metal compositions and the affinities of these enzymes for methanol, ethanol, and other alcohols would need to be examined.

Elucidation of the role of XoxF in this strain is important since many members of the Roseobacter group contain *xoxF* genes. The role of *xoxF* in these marine bacteria warrants further investigation, especially in cultures that are supplemented with lanthanides. Our findings that just under 20% of the Roseobacter genomes examined in this study contain a *xoxF5* suggest that the potential for methylotrophy within this group is larger than previously thought. Since many Roseobacter strains harbor *xoxF5* sequences, this could have important implications for the capacity of the marine environment to act as a sink of methanol and needs to be investigated further, especially since many strains are associated with phytoplankton (Gonzalez et al., 2000; Grossart et al., 2005; Amin et al., 2012, 2015) which have recently been shown to produce high concentrations of methanol. Therefore further work will include investigating the distribution, diversity and activity of such methylotrophs in the marine environment using a variety of cultivation-independent techniques.

16S rRNA gene sequence comparisons place strain La 6 unambiguously within the genus *Marinibacterium*, while overall genome similarities to the type strain *M. profundimaris* 2II1-22F33^T^, determined via digital DDH, were shown to be clearly below the common species cutoff of 70% (Goris et al., 1998; Meier-Kolthoff et al., 2014). Furthermore, the vast differences seen between strain La 6 and its closest neighbors at the MLSA and gene content level clearly demonstrates the need for comparative genomics to be used as a tool to understand the ecological roles and metabolic plasticity of different members of the Roseobacter group. Based on this and the DDH values, we propose that the strain La 6 represents a novel species of the genus *Marinibacterium*. We propose the name *Marinibacterium anthonyi* strain La 6 (in honor of the British microbiologist Professor Christopher Anthony).

## Author Contributions

JM and YC conceived the project. AH conducted all lab work except sequencing, annotation, and comparative genomics, which was conducted by JV and AK-K. JM, CG, MT, JT, and JD provided guidance and insight during the project. AH and JV wrote the manuscript, with all authors providing constructive feedback and approval of the final manuscript.

## Conflict of Interest Statement

The authors declare that the research was conducted in the absence of any commercial or financial relationships that could be construed as a potential conflict of interest.

## Footnotes

1 http://www.bioinformatics.babraham.ac.uk/projects/fastq_screen/

## References

[B1] Alejandro-Marín C. M. Bosch R. Nogales B. (2014). Comparative genomics of the protocatechuate branch of the β-ketoadipate pathway in the Roseobacter lineage. *Mar. Genomics* 17 25–33. 10.1016/j.margen.2014.05.008 24906178

[B2] Amin S. A. Hmelo L. R. van Tol H. M. Durham B. P. Carlson L. T. Heal K. R. (2015). Interaction and signalling between a cosmopolitan phytoplankton and associated bacteria. *Nature* 522 98–101. 10.1038/nature14488 26017307

[B3] Amin S. A. Parker M. S. Armbrust E. V. (2012). Interactions between Diatoms and Bacteria. *Microbiol. Mol. Biol. Rev.* 76 667–684. 10.1128/MMBR.00007-12 22933565 PMC3429620

[B4] Anthony C. (1982). *The Biochemistry of Methylotrophs*, Vol. 75 London: Academic Press.

[B5] Anthony C. (1986). “Bacterial oxidation of methane and methanol,” in *Advances in Microbial Physiology* Vol. 27 eds Rose A. H. Tempest D. W. (London: Academic Press), 113–210. 10.1016/s0065-2911(08)60305-7 3020939

[B6] Beale R. Dixon J. L. Arnold S. R. Liss P. S. Nightingale P. D. (2013). Methanol, acetaldehyde, and acetone in the surface waters of the Atlantic Ocean. *J. Geophys. Res.* 118 5412–5425. 10.1002/jgrc.20322

[B7] Beale R. Liss P. S. Dixon J. L. Nightingale P. D. (2011). Quantification of oxygenated volatile organic compounds in seawater by membrane inlet-proton transfer reaction/mass spectrometry. *Anal. Chim. Acta* 706 128–134. 10.1016/j.aca.2011.08.023 21995919

[B8] Bogart J. A. Lewis A. J. Schelter E. J. (2015). DFT study of the active site of the XoxF-type natural, cerium-dependent methanol dehydrogenase enzyme. *Chemistry* 21 1743–1748. 10.1002/chem.201405159 25421364

[B9] Bolger A. M. Lohse M. Usadel B. (2014). Trimmomatic: a flexible trimmer for illumina sequence data. *Bioinformatics* 30 2114–2120. 10.1093/bioinformatics/btu170 24695404 PMC4103590

[B10] Buchan A. (2001). *Ecology and Genetics of Aromatic Compound Degradation in the Ecologically Important Roseobacter Lineage of Marine Bacteria.* Athens: The University of Georgia.

[B11] Buchan A. González J. M. Moran M. A. (2005). Overview of the marine Roseobacter lineage. *Appl. Environ. Microbiol.* 71 5665–5677. 10.1128/AEM.71.10.5665 16204474 PMC1265941

[B12] Buchan A. Neidle E. L. Moran M. A. (2004). Diverse organization of genes of the β-ketoadipate pathway in members of the marine roseobacter lineage. *Appl. Environ. Microbiol.* 70 1658–1668. 10.1128/AEM.70.3.1658-1668.2004 15006791 PMC368412

[B13] Carrión O. Curson A. R. Kumaresan D. Fu Y. Lang A. S. Mercadé E. (2015). A novel pathway producing dimethylsulphide in bacteria is widespread in soil environments. *Nat. Commun.* 6:6579. 10.1038/ncomms7579 25807229

[B14] Castresana J. (2000). Selection of conserved blocks from multiple alignments for their use in phylogenetic analysis. *Mol. Biol. Evol.* 17 540–552. 10.1093/oxfordjournals.molbev.a026334 10742046

[B15] Chistoserdova L. (2011). Modularity of methylotrophy, Revisited. *Environ. Microbiol.* 13 2603–2622. 10.1111/j.1462-2920.2011.02464.x 21443740

[B16] Chistoserdova L. (2016). Lanthanides: new life metals? *World J. Microbiol. Biotechnol.* 32:138. 10.1007/s11274-016-2088-2 27357406

[B17] Chistoserdova L. Kalyuzhnaya M. G. Lidstrom M. E. (2009). The expanding world of methylotrophic metabolism. *Annu. Rev. Microbiol.* 63 477–499. 10.1146/annurev.micro.091208.073600 19514844 PMC2827926

[B18] Chistoserdova L. Lidstrom M. E. (1997). Molecular and mutational analysis of a DNA region separating two methylotrophy gene clusters in *Methylobacterium extorquens* AM1. *Microbiology* 143 1729–1736. 10.1099/00221287-143-5-1729 9168622

[B19] Cho J. C. Giovannoni S. J. (2006). Pelagibaca bermudensis Gen. Nov., Sp. Nov., a novel marine bacterium within the Roseobacter clade in the order *Rhodobacterales*. *Int. J. Syst. Evol. Microbiol.* 56 855–859. 10.1099/ijs.0.64063-0 16585706

[B20] Choi J. M. Kim H. G. Kim J. S. Youn H. S. Eom S. H. Yu S. L. (2011). Purification, crystallization and preliminary X-ray crystallographic analysis of a methanol dehydrogenase from the marine bacterium *Methylophaga aminisulfidivorans* MPT. *Acta Crystallogr. Sect. F Struct. Biol. Cryst. Commun.* 67 513–516. 10.1107/S1744309111006713 21505255 PMC3080164

[B21] Curson A. R. Liu J. Bermejo Martínez A. Green R. T. Chan Y. Carrión O. (2017). Dimethylsulfoniopropionate biosynthesis in marine bacteria and identification of the key gene in this process. *Nat. Microbiol.* 2:17009. 10.1038/nmicrobiol.2017.9 28191900

[B22] Curson A. R. Todd J. D. Sullivan M. J. Johnston A. W. (2011). Catabolism of Dimethylsulphoniopropionate: microorganisms, enzymes and genes. *Nat. Rev. Microbiol.* 9 849–859. 10.1038/nrmicro2653 21986900

[B23] Dixon J. L. Beale R. Nightingale P. D. (2011). Microbial methanol uptake in northeast atlantic waters. *ISME J.* 5 704–716. 10.1038/ismej.2010.169 21068775 PMC3105731

[B24] Dixon J. L. Beale R. Nightingale P. D. (2013a). Production of methanol, acetaldehyde, and acetone in the atlantic ocean. *Geophys. Res. Lett.* 40 4700–4705. 10.1002/grl.50922

[B25] Dixon J. L. Sargeant S. Nightingale P. D. Colin Murrell J. (2013b). Gradients in microbial methanol uptake: productive coastal upwelling waters to oligotrophic gyres in the Atlantic Ocean. *ISME J.* 7 568–580. 10.1038/ismej.2012.130 23178665 PMC3578572

[B26] Doyle J. J. Doyle J. L. (1987). A rapid DNA isolation procedure for small quantities of fresh leaf tissue. *Phytochem. Bull.* 19 11–15. 10.2307/4119796

[B27] Dziewit L. Czarnecki J. Prochwicz E. Wibberg D. Schlüter A. Pühler A. (2015). Genome-guided insight into the methylotrophy of *Paracoccus aminophilus* JCM 7686. *Front. Microbiol.* 6:852. 10.3389/fmicb.2015.00852 26347732 PMC4543880

[B28] Edgar R. C. (2004). MUSCLE: multiple sequence alignment with high accuracy and high throughput. *Nucleic Acids Res.* 32 1792–1797. 10.1093/nar/gkh340 15034147 PMC390337

[B29] Elderfield H. R. Upstill-Goddard R. Sholkovitz E. R. (1990). The rare earth elements in rivers, estuaries and coastal sea waters: processes affecting crustal input of elements to the ocean and their significance to the composition of sea water. *Geochim. Cosmochim. Acta* 54 971–991. 10.1016/0016-7037(90)90432-K

[B30] Farhan Ul-Haque M. Kalidass B. Bandow N. Turpin E. A. Dispirito A. A. Semrau J. D. (2015). Cerium regulates expression of alternative methanol dehydrogenases in *Methylosinus trichosporium* OB3b. *Appl. Environ. Microbiol.* 81 7546–7552. 10.1128/AEM.02542-15 26296730 PMC4592857

[B31] Figurski D. H. Helinski D. R. (1979). Replication of an origin-containing derivative of plasmid RK2 dependent on a plasmid function provided in trans. *Proc. Natl. Acad. Sci. U.S.A.* 76 1648–1652. 10.1073/pnas.76.4.1648 377280 PMC383447

[B32] Garcia-Solsona E. Jeandel C. Labatut M. Lacan F. Vance D. Chavagnac V. (2014). Rare earth elements and Nd isotopes tracing water mass mixing and particle-seawater interactions in the SE Atlantic. *Geochim. Cosmochim. Acta* 125 351–372. 10.1016/j.gca.2013.10.009

[B33] Giovannoni S. J. Hayakawa D. H. Tripp H. J. Stingl U. Givan S. A. Cho J. C. (2008). The small genome of an abundant coastal ocean methylotroph. *Environ. Microbiol.* 10 1771–1782. 10.1111/j.1462-2920.2008.01598.x 18393994

[B34] Gonzalez J. M. Mayer F. Moran M. A. Hodson R. E. Whitman W. B. (1997). *Sagittula stellata* Gen. Nov., Sp. Nov., a lignin-transforming bacterium from a coastal environment. *Int. J. Syst. Bacteriol.* 47 773–780. 10.1099/00207713-47-3-773 9226910

[B35] Gonzalez J. M. Simó R. Massana R. Covert J. S. Casamayor E. O. Pedrós-Alió C. (2000). Bacterial community structure associated with a Dimethylsulfoniopropionate-producing north Atlantic Algal Bloom. *Appl. Environ. Microbiol.* 66 4237–4246. 10.1128/AEM.66.10.4237-4246.2000 11010865 PMC92291

[B36] Goodwin K. D. Varner R. K. Crill P. M. Oremland R. S. (2001). Consumption of tropospheric levels of methyl bromide by C1 compound-utilizing bacteria and comparison to saturation kinetics. *Appl. Environ. Microbiol.* 67 5437–5443. 10.1128/AEM.67.12.5437-5443.2001 11722890 PMC93327

[B37] Goris J. Suzuki K. De Vos P. Nakase T. Kersters K. (1998). Evaluation of a microplate DNA - DNA hybridization method compared with the initial renaturation method. *Can. J. Microbiol.* 44 1148–1153. 10.1139/w98-118

[B38] Greaves M. J. Rudnicki M. Elderfield H. (1991). Rare earth elements in the mediterranean sea and mixing in the mediterranean outflow. *Earth Planet. Sci. Lett.* 103 169–181. 10.1016/0012-821X(91)90158-E

[B39] Grob C. Taubert M. Howat A. M. Burns O. J. Dixon J. L. Richnow H. H. (2015). Combining metagenomics with metaproteomics and stable isotope probing reveals metabolic pathways used by a naturally occurring marine methylotroph. *Environ. Microbiol.* 17 4007–4018. 10.1111/1462-2920.12935 26033676

[B40] Grossart H. P. Levold F. Allgaier M. Simon M. Brinkhoff T. (2005). Marine diatom species harbour distinct bacterial communities. *Environ. Microbiol.* 7 860–873. 10.1111/j.1462-2920.2005.00759.x 15892705

[B41] Hatje V. Bruland K. W. Flegal A. R. (2014). Determination of rare earth elements after pre-concentration using NOBIAS-chelate PA-1resin: method development and application in the San Francisco Bay Plume. *Mar. Chem.* 160 34–41. 10.1016/j.marchem.2014.01.006

[B42] Howat A. M. (2017). *Characterisation of Novel Methylotrophs and the Role of xoxF in Coastal Marine Environments.* Norwich: University of East Anglia.

[B43] Hu Z. Richter H. Sparovek G. Schnug E. (2004). Physiological and biochemical effects of rare earth elements on plants and their agricultural significance: a review. *J. Plant Nutr.* 27 183–220. 10.1081/PLN-120027555

[B44] Janvier M. Frehel C. Grimont F. Gasser F. (1985). Methylophaga marina Gen. Nov., Sp. Nov. and *Methylophaga thalassica* Sp. Nov., Marine Methylotrophs. *Int. J. Syst. Bacteriol.* 35 131–139. 10.1099/00207713-35-2-131

[B45] Kameyama S. Tanimoto H. Inomata S. Tsunogai U. Ooki A. Takeda S. (2010). High-resolution measurement of multiple volatile organic compounds dissolved in seawater using equilibrator inlet-proton transfer reaction-mass spectrometry (EI-PTR-MS). *Mar. Chem.* 122 59–73. 10.1016/j.marchem.2010.08.003 19791769

[B46] Keltjens J. T. Pol A. Reimann J. Op Den Camp H. J. M. (2014). PQQ-dependent methanol dehydrogenases: rare-earth elements make a difference. *Appl. Microbiol. Biotechnol.* 98 6163–6183. 10.1007/s00253-014-5766-8 24816778

[B47] Kim H. G. Han G. H. Kim D. Choi J. S. Kim S. W. (2012). Comparative analysis of two types of methanol dehydrogenase from *Methylophaga aminisulfidivorans* MP T grown on methanol. *J. Basic Microbiol.* 52 141–149. 10.1002/jobm.201000479 21656818

[B48] Lafay B. Ruimy R. de Traubenberg C. R. Breittmayer V. Gauthier V. Christen R. (1995). Roseobacter algicola Sp. Nov., a new marine bacterium isolated from the phycosphere of the toxin-producing dinoflagellate prorocentrum lima. *Int. J. Syst. Bacteriol.* 45 290–296. 10.1099/00207713-45-2-290 7537061

[B49] Lechner M. Findeiss S. Steiner L. Marz M. Stadler P. F. Prohaska S. J. (2011). Proteinortho: detection of (Co-)Orthologs in large-scale analysis. *BMC Bioinformatics* 12:124. 10.1186/1471-2105-12-124 21526987 PMC3114741

[B50] Lee K. Choo Y. J. Giovannoni S. J. Cho J. C. (2007). *Maritimibacter alkaliphilus* Gen. Nov., Sp. Nov., a genome-sequenced marine bacterium of the Roseobacter clade in the order *Rhodobacterales*. *Int. J. Syst. Evol. Microbiol.* 57 1653–1658. 10.1099/ijs.0.64960-0 17625211

[B51] Li G. Lai Q. Du Y. Liu X. Sun F. Shao Z. (2015). *Marinibacterium profundimaris* Gen. Nov., Sp. Nov., isolated from deep seawater. *Int. J. Syst. Evol. Microbiol.* 65 4175–4179. 10.1099/ijsem.0.000557 26303913

[B52] Luo H. Moran M. A. (2014). Evolutionary ecology of the marine Roseobacter clade. *Microbiol. Mol. Biol. Rev.* 78 573–587. 10.1128/MMBR.00020-14 25428935 PMC4248658

[B53] Magoč T. Salzberg S. L. (2011). FLASH: fast length adjustment of short reads to improve genome assemblies. *Bioinformatics* 27 2957–2963. 10.1093/bioinformatics/btr507 21903629 PMC3198573

[B54] Martens T. Heidorn T. Pukal R. Simon M. Tindall B. J. Brinkhoff T. (2006). Reclassification of *Roseobacter gallaeciensis* Ruiz-Ponte et al. 1998 as phaeobacter gallaeciensis Gen. Nov., Comb. Nov., description of *Phaeobacter inhibens* Sp. Nov., reclassification of *Ruegeria algicola* (Lafay et al., 1995) Uchino et al. 1999 as *Marinovum algicola* Gen. Nov., Comb. Nov., and emended descriptions of the genera *Roseobacter*, *Ruegeria* and *Leisingera*. *Int. J. Syst. Evol. Microbiol.* 56 1293–1304. 10.1099/ijs.0.63724-0 16738106

[B55] McDonald I. R. Murrell J. C. (1997). The methanol dehydrogenase structural gene mxaf and its use as a functional gene probe for methanotrophs and methylotrophs. *Appl. Environ. Microbiol.* 63 3218–3224. 9251208 10.1128/aem.63.8.3218-3224.1997 PMC168619

[B56] Meier-Kolthoff J. P. Klenk H. P. Göker M. (2014). Taxonomic Use of DNA G+C content and DNA-DNA hybridization in the genomic age. *Int. J. Syst. Evol. Microbiol.* 64 352–356. 10.1099/ijs.0.056994-0 24505073

[B57] Mincer T. J. Aicher A. C. (2016). Methanol production by a broad phylogenetic array of marine phytoplankton. *PLoS One* 11:e0150820. 10.1371/journal.pone.0150820 26963515 PMC4786210

[B58] Moran M. A. Reisch C. R. Kiene R. P. Whitman W. B. (2012). Genomic insights into bacterial DMSP transformations. *Annu. Rev. Mar. Sci.* 4 523–542. 10.1146/annurev-marine-120710-100827 22457986

[B59] Nakagawa T. Mitsui R. Tani A. Sasa K. Tashiro S. Iwama T. (2012). A catalytic role of XoxF1 as La3+-dependent methanol dehydrogenase in *Methylobacterium extorquens* Strain AM1. *PLoS One* 7:e50480. 10.1371/journal.pone.0050480 23209751 PMC3507691

[B60] Neufeld J. D. Boden R. Moussard H. Schaefer H. Murrell J. C. (2008a). Substrate-specific clades of active marine methylotrophs associated with a phytoplankton bloom in a temperate coastal environment. *Appl. Environ. Microbiol.* 74 7321–7328. 10.1128/AEM.01266-08 18849453 PMC2592898

[B61] Neufeld J. D. Chen Y. Dumont M. G. Colin Murrell J. (2008b). Marine methylotrophs revealed by stable-isotope probing, multiple displacement amplification and metagenomics. *Environ. Microbiol.* 10 1526–1535. 10.1111/j.1462-2920.2008.01568.x 18294205

[B62] Neufeld J. D. Schäfer H. Cox M. J. Boden R. McDonald I. R. Colin Murrell J. (2007). Stable-isotope probing implicates Methylophaga Spp and novel gammaproteobacteria in marine methanol and methylamine metabolism. *ISME J.* 1 480–491. 10.1038/ismej.2007.65 18043650

[B63] Newton R. J. Griffin L. E. Bowles K. M. Meile C. Gifford S. M. Givens C. E. (2010). Genome characteristics of a generalist marine bacterial lineage. *ISME J.* 4 784–798. 10.1038/ismej.2009.150 20072162

[B64] O’Connell J. Schulz-Trieglaff O. Carlson E. Hims M. M. Gormley N. A. Cox A. J. (2015). NxTrim: optimized trimming of illumina mate pair reads. *Bioinformatics* 31 2035–2037. 10.1093/bioinformatics/btv057 25661542

[B65] Parks D. H. Imelfort M. Skennerton C. T. Hugenholtz P. Tyson G. W. (2015). CheckM: assessing the quality of microbial genomes recovered from isolates, single cells, and metagenomes. *Genome Res.* 25 1043–1055. 10.1101/gr.186072.114 25977477 PMC4484387

[B66] Pol A. Barends T. R. Dietl A. Khadem A. F. Eygensteyn J. Jetten S. M. (2014). Rare earth metals are essential for methanotrophic life in volcanic mudpots. *Environ. Microbiol.* 16 255–264. 10.1111/1462-2920.12249 24034209

[B67] Pradella S. Päuker O. Petersen J. (2010). Genome organisation of the marine roseobacter clade member *Marinovum algicola*. *Arch. Microbiol.* 192 115–126. 10.1007/s00203-009-0535-2 20039020

[B68] Read K. A. Carpenter L. J. Arnold S. R. Beale R. Nightingale P. D. Hopkins J. R. (2012). Multiannual Observations of Acetone, Methanol, and Acetaldehyde in Remote Tropical Atlantic Air: Implications for Atmospheric OVOC Budgets and Oxidative Capacity. *Environ. Sci. Technol.* 46 11028–11039. 10.1021/es302082p 22963451

[B69] Reisch C. R. Moran M. A. Whitman W. B. (2008). Dimethylsulfoniopropionate-dependent demethylase (DmdA) from *Pelagibacter ubique* and *Silicibacter pomeroyi*. *J. Bacteriol.* 190 8018–8024. 10.1128/JB.00770-08 18849431 PMC2593244

[B70] Sambrook J. Russell D. W. (2001). *Molecular Cloning: A Laboratory Manual*, 3rd Edn. New York, NY: Cold Spring Harbor Laboratory Press.

[B71] Schaefer J. K. Goodwin K. D. McDonald I. R. Murrell J. C. Oremland R. S. (2002). *Leisingera methylohalidivorans* Gen. Nov., Sp. Nov., a marine methylotroph that grows on methyl bromide. *Int. J. Syst. Evol. Microbiol.* 52 851–859. 12054249 10.1099/00207713-52-3-851

[B72] Schäfer A. Tauch A. Jager W. Kalinowski J. Thierbach G. Puhler A. (1994). Small mobilizable multi-purpose cloning vectors derived from the *Escherichia Coli* plasmids pK18 and pK19: selection of defined deletions in the chromosome of corynebacterium glutamicum. *Gene* 145 69–73. 10.1016/0378-1119(94)90324-7 8045426

[B73] Schäfer H. McDonald I. R. Nightingale P. D. Murrell J. C. (2005). Evidence for the presence of a CmuA methyltransferase pathway in novel marine methyl halide-oxidizing bacteria. *Environ. Microbiol.* 7 839–852. 10.1111/j.1462-2920.2005.00757.x 15892703

[B74] Schäfer H. Myronova N. Boden R. (2010). Microbial degradation of dimethylsulphide and related C1-sulphur compounds: organisms and pathways controlling fluxes of sulphur in the biosphere. *J. Exp. Bot.* 61 315–334. 10.1093/jxb/erp355 20007683

[B75] Schmieder R. Edwards R. (2011). Quality control and preprocessing of metagenomic datasets. *Bioinformatics* 27 863–864. 10.1093/bioinformatics/btr026 21278185 PMC3051327

[B76] Seemann T. (2014). Prokka: rapid prokaryotic genome annotation. *Bioinformatics* 30 2068–2069. 10.1093/bioinformatics/btu153 24642063

[B77] Seo J. S. Keum Y. S. Li Q. X. (2009). Bacterial degradation of aromatic compounds. *Int. J. Environ. Res. Public Health* 6 278–309. 10.3390/ijerph6010278 19440284 PMC2672333

[B78] Shiba T. (1991). *Roseobacter litoralis* Gen. Nov., Sp. Nov., and *Roseobacter denitrificans* Sp. Nov., aerobic pink-pigmented bacteria which contain Bacteriochlorophyll a. *Syst. Appl. Microbiol.* 14 140–145. 10.1016/S0723-2020(11)80292-4

[B79] Simon M. Scheuner C. Meier-Kolthoff J. P. Brinkhoff T. Wagner-Döbler I. Ulbrich M. (2017). Phylogenomics of rhodobacteraceae reveals evolutionary adaptation to marine and non-marine habitats. *ISME J.* 11 1483–1499. 10.1038/ismej.2016.198 28106881 PMC5437341

[B80] Strand S. E. Lidstrom M. E. (1984). Characterization of a new marine methylotroph. *FEMS Microbiol. Lett.* 21 247–251. 10.1111/j.1574-6968.1984.tb00219.x

[B81] Studer A. Stupperich E. Vuilleumier S. Leisinger T. (2001). Chloromethane: tetrahydrofolate methyl transfer by two proteins from *Methylobacterium chloromethanicum* strain CM4. *Eur. J. Biochem.* 268 2931–2938. 10.1046/j.1432-1327.2001.02182.x 11358510

[B82] Studer A. McAnulla C. Büchele R. Leisinger T. Vuilleumier S. (2002). Chloromethane-induced genes define a third C_1_ utilization pathway in *Methylobacterium chloromethanicum* CM4. *J. Bacteriol.* 184 3476–3484. 10.1128/JB.184.13.3476-3484.2002 12057941 PMC135114

[B83] Sun F. Wang B. Liu X. Lai Q. Du Y. Li G. (2010). *Leisingera nanhaiensis* Sp.nov., isolated from marine sediment. *Int. J. Syst. Evol. Microbiol.* 60 275–280. 10.1099/ijs.0.010439-0 19651744

[B84] Sun J. Todd J. D. Thrash J. C. Qian Y. Qian M. C. Temperton B. (2016). The abundant marine bacterium *Pelagibacter* simultaneously catabolizes Dimethylsulfoniopropionate to the gases dimethyl sulfide and methanethiol. *Nat. Microbiol.* 1:16065. 10.1038/nmicrobiol.2016.65 27573103

[B85] Taubert M. Grob C. Howat A. M. Burns O. J. Dixon J. L. Chen Y. (2015). XoxF encoding an alternative methanol dehydrogenase is widespread in coastal marine environments. *Environ. Microbiol.* 17 3937–3948. 10.1111/1462-2920.12896 25943904

[B86] Tett A. J. Rudder S. J. Bourdès A. Karunakaran R. Poole P. S. (2012). Regulatable vectors for environmental gene expression in alphaproteobacteria. *Appl. Environ. Microbiol.* 78 7137–7140. 10.1128/AEM.01188-12 22820336 PMC3457481

[B87] Todd J. D. Curson A. R. Kirkwood M. Sullivan M. J. Green R. T. Johnston A. W. (2011). DddQ, a novel, cupin-containing, dimethylsulfoniopropionate lyase in marine Roseobacters and in uncultured marine bacteria. *Environ. Microbiol.* 13 427–438. 10.1111/j.1462-2920.2010.02348.x 20880330

[B88] Vorholt J. A. Marx C. J. Lidstrom M. E. Thauer R. K. (2000). Novel formaldehyde-activating enzyme in *Methylobacterium extorquens* AM1 required for growth on methanol. *J. Bacteriol.* 182 6645–6650. 10.1128/JB.182.23.6645-6650.2000 11073907 PMC111405

[B89] Vu H. N. Subuyuj G. A. Vijayakumar S. Good N. M. Martinez-Gomez N. C. Skovran E. (2016). Lanthanide-dependent regulation of methanol oxidation systems in *Methylobacterium extorquens* AM1 and their contribution to methanol growth. *J. Bacteriol.* 198 1250–1259. 10.1128/JB.00937-15 26833413 PMC4859578

[B90] Wagner-Döbler I. Biebl H. (2006). Environmental biology of the marine Roseobacter lineage. *Annu. Rev. Microbiol.* 60 255–280. 10.1146/annurev.micro.60.080805.142115 16719716

[B91] Williams J. Holzinger R. Gros V. Xu V. Atlas E. Wallace D. W. R. (2004). Measurements of organic species in air and seawater from the tropical Atlantic. *Geophys. Res. Lett.* 31:L23S06 10.1029/2004GL020012

[B92] Wilson S. M. Gleisten M. P. Donohue T. J. (2008). Identification of proteins involved in formaldehyde metabolism by *Rhodobacter sphaeroides*. *Microbiology* 154 296–305. 10.1099/mic.0.2007/011346-0 18174148 PMC2440690

[B93] Wu M. L. Wessels J. C. Pol A. Op den Camp H. J. Jetten M. S. van Niftrik L. (2015). XoxF-type methanol dehydrogenase from the Anaerobic methanotroph ‘Candidatus *Methylomirabilis oxyfera.*’ *Appl. Environ. Microbiol.* 81 1442–1451. 10.1128/AEM.03292-14 25527536 PMC4309699

[B94] Yamamoto M. Y. Kouno S. K. Okamoto R. Inui T. (1978). Isolation and characterization of marine methanol-utilizing bacteria. *J. Ferment. Technol.* 56 451–458. 10.1016/j.micres.2013.04.002 23632047

