# Supplementary material for: Comparative Genomics and Mutational Analysis Reveals a Novel XoxF-Utilizing Methylotroph in the Roseobacter Group Isolated From the Marine Environment
Source: Front Microbiol. 2018 Apr 27;9:766. doi: 10.3389/fmicb.2018.00766 (PMC5934484; doi:10.3389/fmicb.2018.00766)
Supplement: Supplementary file 1 [file Data_Sheet_1.DOCX]

Supplementary information

**Comparative genomics and mutational analysis reveals a novel XoxF-utilising methylotroph in the Roseobacter group isolated from a marine environment**

Alexandra M. Howat^1^, John Vollmers^2^, Martin Taubert^3^, Carolina Grob^1^, Joanna L. Dixon^4^, Jonathan Todd^5^, Yin Chen^6^, Anne-Kristin Kaster^2^, J. Colin Murrell^1*^

^1^School of Environmental Sciences, University of East Anglia, Norwich, UK

^2^Institute for Biological Interfaces (IGB 5), Karlsruhe Institute of Technology, Germany

^3^Aquatic Geomicrobiology, Institute of Biodiversity, Friedrich Schiller University Jena, Jena, Germany

^4^Plymouth Marine Laboratory, Plymouth, UK

^5^School of Biological Sciences, University of East Anglia, Norwich, UK

^6^School of Life Sciences, University of Warwick, Coventry, UK

Correspondence:

Prof. J. Colin Murrell

Email: [j.c.murrell@uea.ac.uk](mailto:j.c.murrell@uea.ac.uk)

**Supplementary Figures**

Supplementary Figure 1: Concentration of methanol in the headspace of methanol enrichments set up using seawater samples from station L4, Plymouth, with or without the Rare Earth Elements (REEs) lanthanum (La) and cerium (Ce), and incubated over a period of 12 days. All enrichments contained methanol (shown) except control vials containing both REEs but no methanol, which were only measured on the GC on the first and last days (not shown). Data points represent triplicate vials; error bars indicate the standard error. REEs were added in the form of chloride heptahydrate salts.

^^

Supplementary Figure 2: Phylogenetic analysis of 16S rRNA gene sequences of strain La 6 and members of the Roseobacter group. Evolutionary distance among 16S rRNA gene sequences of strain La 6 and all type strains of the species within the Roseobacter group is illustrated by a Neighbour-joining tree. 16S sequences were aligned against the SILVA database using SINA (Pruesse, Peplies, and Glöckner 2012), and clustered using the Neighbour-joining algorithm with 1000 bootstrap permutations as implemented by ARB v. 6.0.2 (Ludwig et al. 2004).

^
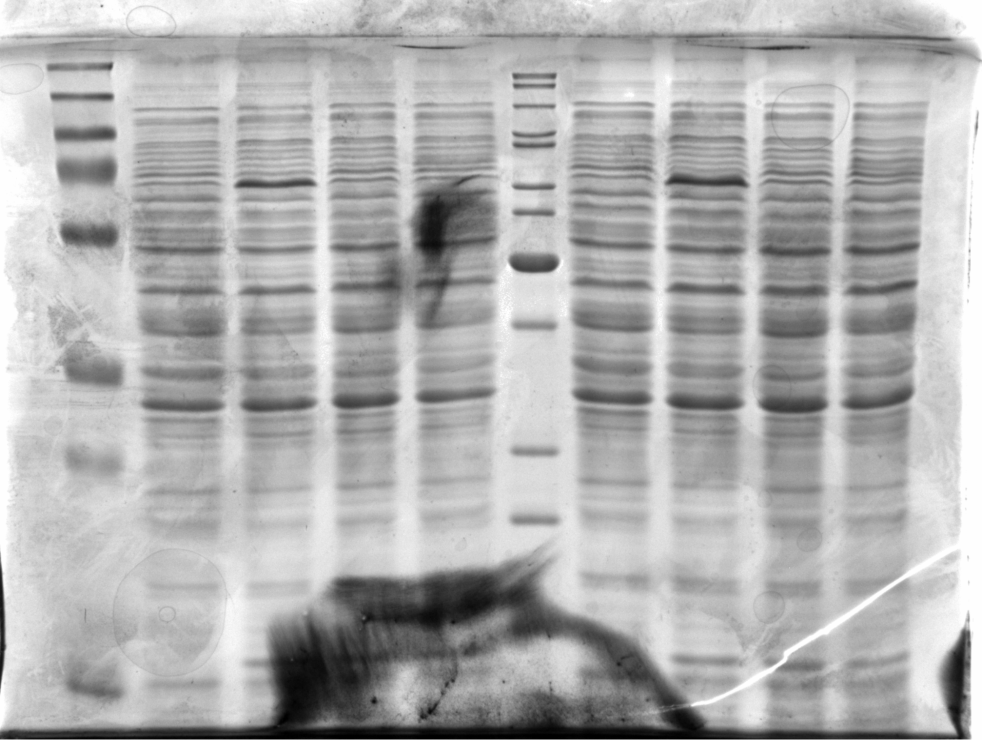

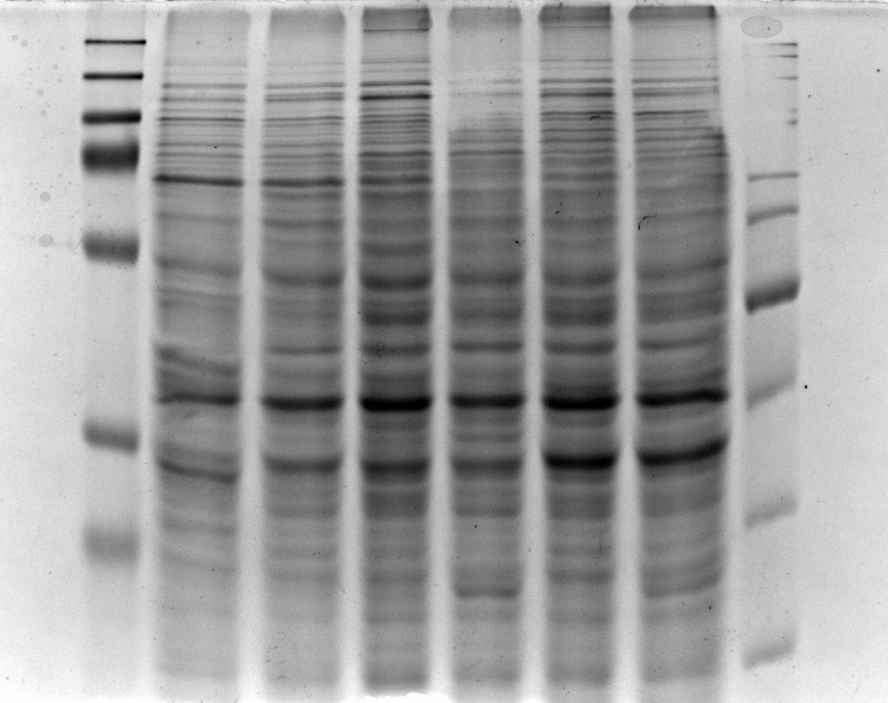
^

**A B**

M wt wt wt XoxF^-^ wt XoxF^-^

M E S S B B

130

100

kDa

70

55

35

25

M XoxF^-^ XoxF^-^ p509LA6

S+M S+M

50

60

70

40

30

25

kDa

85

Supplementary Figure 3: SDS-PAGE of mutant strains XoxF^-^ and XoxF^-^ p509LA6 grown on different carbon sources. A) Strain XoxF^-^ grown on succinate and benzoate, showing the missing XoxF band compared to the presence in the wild type (wt) grown on methanol, ethanol, succinate and benzoate. B) Strain XoxF^-^ p509LA6 showing restored expression of XoxF when grown on succinate and methanol compared to the mutant XoxF^-^. Abbreviations: M, methanol; E, ethanol; S, succinate; B, benzoate. The band corresponding to the XoxF polypeptide is indicated by the arrow (confirmed by mass spectrometry analysis of this polypeptide).

Supplementary Figure 4: Growth of strain La 6 wild-type strain (circles), complemented strain La 6 XoxF^-^ p509LA6 (triangles), control strain XoxF^−^ pLMB509 (crosses) and no-inoculum controls (squares) on 5 mM methanol (left), ethanol (right, dark grey) and succinate (right, light grey). Error bars show the range of duplicate cultures.

**Supplementary Tables**

**Supplementary Table 1:** List of organisms, plasmids and primers used in this study.

|  | **Description/genotype** | **Reference/source** |
| --- | --- | --- |
| **Strains** |  |  |
| La 6 | Wild-type strain | This study |
| La 6^Rif^ | Wild-type strain, Rif^R^ | This study |
| La 6 XoxF^−^ | La 6^Rif^ *XoxF::*p672*xoxF*, Km^R^ | This study |
| XoxF^−^ p509LA6 | La 6 XoxF^−^ complemented with the wild-type *xoxF5* gene on | This study |
|  | plasmid p509LA6, Gm^R^ |  |
| XoxF^−^ pLMB509 | La 6 XoxF^−^ carrying the empty expression vector pLMB509, Gm^R^ | This study |
| **Plasmids** |  |  |
| pRK2013 | Km^R^, RK2 vector, self transmissible, helper plasmid | (Figurski and Helinski 1979) |
| pK19*mob* | Km^R^, RP4-mob, mobilizable cloning vector | (Schafer et al. 1994) |
| p672*xoxF* | pK19*mob* containing a 672bp internal fragment of *xoxF* from La 6 | This study |
| pLMB509 | Gm^R^ expression vector with inducible taurine promoter (tauAP); | (Tett et al. 2012) |
|  | *gfp* excised |  |
| p509LA6 | pLMB509 containing *xoxF5* from strain La 6 | This study |
| **Primers** |  |  |
| CheckmutF | 5’- CACCGTGGTGGCGCTGGATGC - 3’ | This study |
| CheckmutR | 5’ **-** ACCCAAGCGGCCGGAGAACCT - 3’ |  |

Supplementary Table 2: Compounds utilised by strain La 6 as sole source of carbon and energy, presented in alphabetical order. Inoculum used was grown on succinate.

| **Carbon source** | **Concentration** | **OD_540_** | **Carbon source** | **Concentration** | **OD_540_** |
| --- | --- | --- | --- | --- | --- |
| 1-butanol | 0.05 % (v/v) | 0.82 | Fructose | 5 mM | 1.20 |
| 1-propanol | 0.05 % (v/v) | 0.28 | Glucose | 5 mM | 1.20 |
| 2-propanol | 0.05 % (v/v) | 0.07 | Glycerol | 5 mM | 0.13 |
| 4-hydroxybenzoate | 3.6 mM | 0.20 | Glycine betaine | 10 mM | 0.13 |
| Acetate | 5 mM | 0.37 | Malate | 5 mM | 0.71 |
| Acetone | 0.05 % (v/v) | 0.13 | Mannitol | 5 mM | 0.76 |
| Acrylic acid | 10 mM | 0.17 | Mannose | 5 mM | 0.96 |
| Alanine | 0.2 % (w/v) | 0.28 | Methanol | 5 mM | 0.18 |
| Arabinose | 5 mM | 0.50 | Propanal | 5 mM | 0.30 |
| Arginine | 0.2 % (w/v) | 0.24 | Propane | 20 % (v/v) | 0.05 |
| Benzoate | 5 mM | 1.00 | Propionate | 5 mM | 0.42 |
| Butane | 20 % (v/v) | 0.17 | Protocatechuate | 5 mM | 0.87 |
| Catechol | 5 mM | 1.32 | Pyruvate | 5 mM | 0.40 |
| Citrate | 5 mM | 0.49 | Ribose | 5 mM | 0.18 |
| Ethanol | 5 mM | 0.54 | Serine | 0.1 % (w/v) | 0.10 |
| Formate | 5 mM | 0.08 | Sorbitol | 5 mM | 0.82 |
| Formate | 20 mM | 0.13 | Succinate | 5 mM | 0.80 |

Strain La 6 was unable to utilise the following carbon compounds as sole source of carbon and energy, in alphabetical order: 2-butanol (0.05%), 3-hydroxybenzoate (3.6 mM), 4-chlorobenzoate (saturated solution), benzene (1 mM), cysteic acid (10 mM), dimethylamine (10 mM), dimethylsulfide (2 mM), dimethylsulfonioproprionate (2 mM), dimethylsulfoxide (0.5 mM), ethane (20 % v/v), glycine (5 mM), glyoxylate (5 mM), lactose (5 mM), methane (20 % v/v), methane sulfonic acid (20 mM), methionine (0.2%), monomethylamine (10 mM), naphthalene (saturated solution), p-cresol (1 mM), p-xylene (1 mM), phenol (0.04%), rhamnose (5 mM), sucrose (5 mM), taurine (10 mM), toluene (0.5 mM), trimethylamine (10 mM), trimethylamine N-oxide (30 mM), urea (5 mM), vanillate (saturated solution).

**Supplementary Table 3**: Cellular fatty acid content of strain La 6 and its closest relative at the 16S rRNA gene level, *Marinibacterium profundimaris* 22II1-22F33 (Li et al. 2015). Values are percentages of total fatty acids. -, not detected.

| **Fatty acid** | ***La 6*** | ***M. profundimaris* 22II1-22F33** |
| --- | --- | --- |
| Summed feature 2* | 7.31 | - |
| 15 : 0 3-OH | 0.87 | - |
| Summed feature** | - | - |
| 16 : 0 | 5.36 | 2.4 |
| 16 : 0 2-OH | 6.19 | 5.6 |
| 16 : 1 2-OH | 1.13 | 2.4 |
| 18 : 0 | 1.16 | 1.4 |
| 18 : 1ω7c/ω6c | 67.83 | 73.8 |
| 18 : 1 ω7c 11-methyl | 6.71 | 5.5 |
| 18: 1 2-OH | - | 3.7 |
| 19 : 0 cyclo w8c | 3.44 | 1.9 |

*14 : 0 3-OH/16:1 iso I or 16:1 iso I /14 : 0 3-OH

** 15 : 0 iso 2-OH / 16 : 1ω7c

**Supplementary Methods**

**Phenotypic characterisation**

Cell cultures were regularly examined under 1,000x magnification in phase-contrast to assess morphology. Gram staining was performed as described by Gerhardt et al. (1994). Utilisation of different carbon sources was tested by monitoring cell density increases in OD_540_ in duplicate compared to controls with no inoculum at 25˚C, shaking at 150 rpm. Cultures were grown for a minimum of 8 days. Sensitivity to antibiotics was tested by two methods: inoculating in 10 ml MB media and monitoring for cell growth by OD_540_ and by using the disc method (Cho and Giovannoni 2003). Growth at different temperatures, pH and salinity were monitored by increases in OD_540_ in triplicate compared to controls with no inoculum for 8 days. Temperatures of 4, 8, 10, 15, 20, 25, 30, 37, 40, 42 and 45˚C in MB were tested. Growth at different pH was tested in MB at every pH increase of 0.5 between 3.5-10, using 10% (v/v) HCl and 10 M NaOH to adjust the pH. Each pH medium was used to blank its respective test condition. Growth at different NaCl concentrations was tested using an artificial salt water (ASW) media, as described by Cho & Giovannoni 2003.

MB plates containing 0.3%, 0.5% and 1% (w/v) agar were used to test for swarming motility, swimming motility and twitching motility, respectively. Cells were also examined under 1000x magnification using phase-contrast for motility in MB and MBM media.

Catalase, oxidase and gelatinase activity were tested as done by Smibert, R & Kreig 1994. The ability to metabolise glucose and lactose by fermentation or oxidation was tested using Hugh and Leifson’s OF basal medium (Hugh and Leifson 1953), with replacement of the NaCl with Sea Salts (Sigma-Aldrich). The ability to reduce nitrate or nitrite was tested using Griess’ reagents (Smibert, R and Kreig 1994), with nitrate/nitrite broth supplemented with Sea Salts. Indole acetic acid production was tested by the method of Glickmann & Dessaux (1995). Oxidation of thiosulfate was performed as described by González et al. (2003). The production of bacteriochlorophyll *a* or other pigments was investigated using the method of Shiba et al. (1991).

Cellulose degradation was tested as described by Kauri & Kushner (1985); MBM plates contained both 10 mM succinate and 0.5% Avicel (microcrystalline cellulose, type PH-105; FMC Corporation) or only Avicel as the carbon source. Half of the plates then had roughly 3 mm of MBM agar (no carbon source) poured on top. *Sagittula stellata* E-37 was used as a positive control. The same method was performed for xylanase activity, using 0.5% birchwood xylan (Sigma), but with no overlaid agar plates. Amylase activity was assayed by streaking a colony onto MB agar plates containing 0.5% soluble starch (Sigma-Aldrich), incubating as previously mentioned and flooding the plate with Gram’s iodine solution. All plates were incubated for 7 days at 30˚C.

Fatty acid analyses were carried out by the Identification Service of the DSMZ, Braunschweig, Germany using the Sherlock Microbial Identification System.

**Measurement of substrates**

Headspace methanol was measured by gas chromatography (GC) on an Agilent 7890A instrument, using a flame ionisation detector, a Porapak Q column (30 m x 0.530 mm, 40 μM film) and nitrogen carrier gas. Standards were prepared in sterile water in the same volume and vials as the relevant experiment.

Measurement of DMSP production and consumption, and production of DMS were performed as described in Carrión et al. 2015 and Curson et al. 2011, respectively.

**NAD(P)-independent alcohol dehydrogenase assay**

Methanol dehydrogenase-like activity was assayed using the artificial electron acceptor phenazine methosulfate (PMS) coupled to the reduction of dichlorophenolindophenol (DCPIP) as described by Anthony & Zatman (1964). Unless otherwise stated, reactions (1 ml) contained Tris buffer (pH 9, 100 mM), PMS (1 mM), DCPIP (0.08 mM), NH_4_Cl (15 mM), protein, and substrate (typically 10 mM). Reactions were initiated by the addition of ammonium and followed spectrophotometrically at 600 nm, using water as a blank. Reactions lacking protein, ammonium or substrate were also performed as controls. Significant transient activity occurred without substrate and was not subtracted from the substrate-induced activity (Day and Anthony 1990). Cell extract was kept on ice at all times and used for no longer than 3 hours. Activity was calculated using ε_600_ (molar extinction coefficient at 600nm) = 1.91 x 10^4^ M^-1^ cm^-1^ for DCPIP (Basford and Huennekens 1955).

**References**

Anthony, C, and L J Zatman. 1964. “The Microbial Oxidation of Methanol. 2. The Methanol-Oxidizing Enzyme of *Pseudomonas* Sp. M 27.” *Biochemical Journal* 92: 614–21.

Basford, R. E., and F. M. Huennekens. 1955. “Studies on Thiols. I. Oxidation of Thiol Groups by 2, 6-Dichlorophenol Indophenol1.” *Journal of the American Chemical Society* 2609 (13): 3873–77. doi:10.1021/ja01619a058.

Carrión, O., a. R. J. Curson, D. Kumaresan, Y. Fu, a. S. Lang, E. Mercadé, and J. D. Todd. 2015. “A Novel Pathway Producing Dimethylsulphide in Bacteria Is Widespread in Soil Environments.” *Nature Communications* 6: 6579. doi:10.1038/ncomms7579.

Cho, Jang Cheon, and Stephen J. Giovannoni. 2003. “*Parvularcula bermudensis* Gen. Nov., Sp. Nov., a Marine Bacterium That Forms a Deep Branch in the α-Proteobacteria.” *International Journal of Systematic and Evolutionary Microbiology* 53 (4): 1031–36. doi:10.1099/ijs.0.02566-0.

Curson, Andrew R. J., Jonathan D. Todd, Matthew J. Sullivan, and Andrew W. B. Johnston. 2011. “Catabolism of Dimethylsulphoniopropionate: Microorganisms, Enzymes and Genes.” *Nature Reviews Microbiology* 9 (12): 849–59. doi:10.1038/nrmicro2653.

Day, D, and Christopher Anthony. 1990. “Methanol Dehydrogenase from *Methylobacterium extorquens* AM1.” *Methods in Enzymology* 188: 210–16.

Figurski, D H, and D R Helinski. 1979. “Replication of an Origin-Containing Derivative of Plasmid RK2 Dependent on a Plasmid Function Provided in Trans.” *Proceedings of the National Academy of Sciences of the United States of America* 76 (4): 1648–52. doi:10.1073/pnas.76.4.1648.

Gerhardt, Phillipp, R. G. E Murray, Willis. A Wood, and Noel. R Kreig. 1994. *Methods for General and Molecular Bacteriology*. Edited by R. G. E Murray, Willis. A Wood, and Noel. R Kreig. Washington DC: American Society for Microbiology.

Glickmann, Eric, and Yves Dessaux. 1995. “A Critical Examination of the Specificity of the Salkowski Reagent for Indolic Compounds Produced by Phytopathogenic Bacteria.” *Appl. Environ. Microbiol.* 61 (2): 793–96.

González, José M., Joseph S. Covert, William B. Whitman, James R. Henriksen, Frank Mayer, Birgit Scharf, Rüdiger Schmitt, et al. 2003. “Silicibacter Pomeroyi Sp. Nov. and *Roseovarius nubinhibens* Sp. Nov., Dimethylsulfoniopropionate-Demethylating Bacteria from Marine Environments.” *International Journal of Systematic and Evolutionary Microbiology* 53 (5): 1261–69. doi:10.1099/ijs.0.02491-0.

Hugh, R., and E. Leifson. 1953. “The Taxonomic Significance of Fermentative versus Oxidative Metabolism of Carbohydrates by Various Gram Negative Bacteria.” *Journal of Bacteriology* 66 (1): 24–26.

Kauri, T, and D J Kushner. 1985. “Role of Contact in Bacterial-Degradation of Cellulose.” *Fems Microbiology Ecology* 31 (5): 301–6. doi:DOI 10.1111/j.1574-6968.1985.tb01163.x.

Li, Guizhen, Qiliang Lai, Yaping Du, Xiupian Liu, Fengqin Sun, and Zongze Shao. 2015. “*Marinibacterium profundimaris* Gen. Nov., Sp. Nov., Isolated from Deep Seawater.” *International Journal of Systematic and Evolutionary Microbiology*, no. 2015: 4175–79. doi:10.1099/ijsem.0.000557.

Ludwig, Wolfgang, Oliver Strunk, Ralf Westram, Lothar Richter, Harald Meier, A. Yadhukumar, Arno Buchner, et al. 2004. “ARB: A Software Environment for Sequence Data.” *Nucleic Acids Research* 32 (4): 1363–71. doi:10.1093/nar/gkh293.

Pruesse, Elmar, Jörg Peplies, and Frank Oliver Glöckner. 2012. “SINA: Accurate High-Throughput Multiple Sequence Alignment of Ribosomal RNA Genes.” *Bioinformatics* 28 (14): 1823–29. doi:10.1093/bioinformatics/bts252.

Schafer, Andreas, Andreas Tauch, Wolfgang Jager, Jorn Kalinowski, Georg Thierbach, and Alfred Puhler. 1994. “Small Mobilizable Multi-Purpose Cloning Vectors Derived from the Escherichia Coli Plasmids pK18 and pK19: Selection of Defined Deletions in the Chromosome of *Corynebacterium glutamicum*.” *Gene* 145 (1): 69–73. doi:10.1016/0378-1119(94)90324-7.

Shiba, T, Y Shioi, K Takamiya, D C Sutton, and C R Wilkinson. 1991. “Distribution and Physiology of Aerobic-Bacteria Containing Bacteriochlorophyll Alpha on the East and West Coasts of Australia.” *Applied and Environmental Microbiology* 57 (1): 295–300. doi:10.1111/lam.12154.

Smibert, R, M, and Noel. R Kreig. 1994. “Phenotypic Characterisation.” In *Methods for General and Molecular Bacteriology*, edited by Phillipp Gerhardt, R. G. E Murray, Willis. A Wood, and Noel. R Kreig, 611–51. Washington, DC: American Society for Microbiology.

Tett, Adrian J., Steven J. Rudder, Alexandre Bourdès, Ramakrishnan Karunakaran, and Philip S. Poole. 2012. “Regulatable Vectors for Environmental Gene Expression in *Alphaproteobacteria*.” *Applied and Environmental Microbiology* 78 (19): 7137–40. doi:10.1128/AEM.01188-12.
